# Brexanolone, a neurosteroid antidepressant, vindicates the GABAergic deficit hypothesis of depression and may foster resilience

**DOI:** 10.12688/f1000research.18758.1

**Published:** 2019-05-29

**Authors:** Bernhard Lüscher, Hanns Möhler

**Affiliations:** 1 Department of Biology and Department of Biochemistry and Molecular Biology, Pennsylvania State University, University Park, PA, 16802, USA; 2 Center for Molecular Investigation of Neurological Disorders, The Huck Institutes for the Life Sciences, Pennsylvania State University, University Park, PA, 16802, USA; 3 Institute of Pharmacology and Neuroscience Center, University of Zurich, Zurich, 8057, Switzerland; 4 Department of Chemistry and Applied Biosciences, Swiss Federal Institute of Technology (ETH), Zurich, 8057, Switzerland

**Keywords:** Major depressive disorder, anxiety, postpartum depression, rapid acting antidepressant, allopregnanolone, neurosteroid, GABA receptor

## Abstract

The GABAergic deficit hypothesis of depression states that a deficit of GABAergic transmission in defined neural circuits is causal for depression. Conversely, an enhancement of GABA transmission, including that triggered by selective serotonin reuptake inhibitors or ketamine, has antidepressant effects. Brexanolone, an intravenous formulation of the endogenous neurosteroid allopregnanolone, showed clinically significant antidepressant activity in postpartum depression. By allosterically enhancing GABA _A_ receptor function, the antidepressant activity of allopregnanolone is attributed to an increase in GABAergic inhibition. In addition, allopregnanolone may stabilize normal mood by decreasing the activity of stress-responsive dentate granule cells and thereby sustain resilience behavior. Therefore, allopregnanolone may augment and extend its antidepressant activity by fostering resilience. The recent structural resolution of the neurosteroid binding domain of GABA _A_ receptors will expedite the development of more selective ligands as a potential new class of central nervous system drugs.

## Introduction

Major depressive disorder (MDD) represents the most common cause of total psychophysiological disability with a worldwide lifetime prevalence of 12 to 20% and estimated annual costs to the US economy of more than $100 billion ^1– 3^. MDD is difficult to treat, in part because it is a phenotypically and etiologically heterogeneous syndrome ^4^. Accordingly, it is challenging to conceive of a single mechanism that could account for most forms of this disease and of a treatment that might alleviate symptoms in the majority of patients. Indeed, current first-line antidepressants that are designed to modulate monoaminergic transmitter systems separate from placebo in only about 50% of clinical trials ^5^, are effective in fewer than two thirds of patients subjected to one or two regimens of treatment ^6^, and act with a delay of several weeks to months ^7^. Even among patients who respond to these treatments, only a small fraction show remission. These features often lead to endless and futile pursuits of an effective treatment and illustrate the enormous unmet need for better antidepressant therapies. Here, we summarize the GABAergic deficit hypothesis of depression and its clinical support by the neurosteroid brexanolone, which largely acts by enhancing GABAergic inhibition.

## The GABAergic deficit hypothesis of depression

The GABAergic deficit hypothesis of MDD posits that diverse defects in GABAergic neural inhibition can causally contribute to common phenotypes of MDD and conversely that the efficacy of current and future antidepressant therapies depends on their ability to restore GABAergic neurotransmission ^8, 9^. Consistent with this hypothesis, clinical studies over the past 15 years have provided compelling evidence that MDD is associated with diverse defects in GABAergic neurotransmission. This includes well-replicated findings of reduced brain levels of GABA ^10– 12^, reduced expression of glutamic acid decarboxylase (GAD) as the principal enzyme responsible for GABA synthesis by GABAergic interneurons ^13, 14^, reduced density or function of GABAergic interneurons ^15– 17^, and reduced expression and function of the principal receptors for GABA known as GABA _A_ receptors ^18– 20^. Together, these changes explain the marked *functional* defects in cortical GABAergic inhibition observed in patients with MDD ^21^.

Beyond MDD, GABAergic deficits are also broadly implicated in anxiety disorders, which are highly comorbid with MDD ^22^ but may have distinct developmental origins ^23^. Compared with other neuropsychiatric disorders, MDD shows low heritability of about 38% ^24^. Even this low heritability remains unexplained as attempts to replicate the identification of candidate genes of MDD have been failing ^25, 26^. Therefore, rather than relying on genetic models to explore disease mechanism, pre-clinical models of MDD are often based on the notion that chronic stress represents a major environmental vulnerability and precipitating factor of MDD. Consistent with a causative role of stress for MDD, chronic exposure of rodents to stress results in diverse behavioral alterations in a direction opposite to those induced by antidepressant drug treatment, and antidepressant drug treatments prevent or ameliorate the detrimental effects of stress in these models ^27, 28^. Chronic stress also results in reduced production and survival of adult-born hippocampal granule cell neurons and these cells are essential for at least some of the behavioral actions of antidepressants ^29^. Importantly, stress-induced behavioral alterations of rodents are associated with impairment of GABAergic interneurons, reduced expression of GAD and of the vesicular and plasma membrane transporters for GABA, and reduced density and function of GABAergic synapses ^30– 34^. In addition, chronic stress leads to marked deficits in the synthesis of endogenous GABA-potentiating neurosteroids, as detailed below. Lastly, chronic stress also leads to a shift in the chloride reversal potential to more depolarized membrane potentials, which renders GABAergic inhibition ineffective ^35, 36^. In corticotropin-releasing hormone (CRH) neurons of the hypothalamus, corresponding stress-induced loss of inhibitory drive leads to chronic hypothalamic–pituitary–adrenal (HPA) axis activation ^35^. Thus, stress-induced defects in GABAergic inhibition are self-perpetuating because they exacerbate stress-induced glutamate release and lead to chronically dysregulated stress axis function. Conversely, mechanisms that enhance GABAergic inhibition are predicted to confer stress resilience, a process that has been described by the American Psychological Association as “adapting well in the face of adversity, trauma, tragedy threats or significant sources of stress” (American Psychological Association, www.apa.org/helpcenter/road-resilience, last checked April 25, 2019).

Some of the most compelling evidence that defects in GABA transmission can *causally* contribute to stress-induced anxiety- and depressive-like symptoms is available from analyses of GABA _A_ receptor mutant mice. Knockout mice that were rendered heterozygous for the γ2 subunit (γ2 ^+/−^ mice, lacking one of 38 gene alleles that contribute to heteropentameric GABA _A_ receptors) exhibit anxiety- and depression-related behavior, defects in hippocampal neurogenesis, cognitive deficits in emotional pattern separation, and chronic HPA axis activation that are expected of an animal model of MDD ^23, 37– 40^. Some of these same behavioral defects have been described in mice lacking the α2 subunit of GABA _A_ receptors ^41^ or the neurosteroid binding site of α2 GABA _A_ receptors ^42^ and in mice with genetically reduced GABA synthesis ^43^.

**Figure 1.**
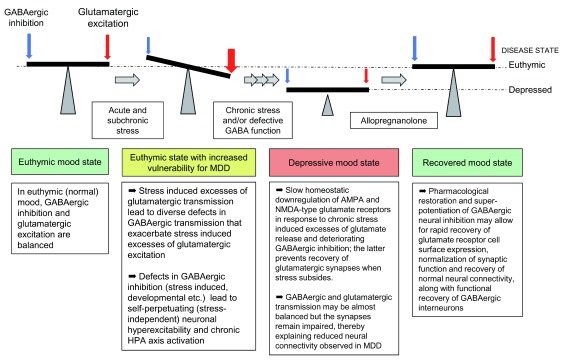
Schematic of chronic stress and GABAergic deficit-induced downregulation of glutamatergic transmission and recovery by allopregnanolone. HPA, hypothalamic–pituitary–adrenal; MDD, major depressive disorder.

Chronic stress and defects in GABAergic transmission of γ2 ^+/−^ mice further have in common that they result in similar homeostatic-like downregulation of ionotropic glutamate receptors (AMPA and NMDA receptors) and glutamatergic synaptic transmission ( Figure 1) ^44– 46^. The anxious and depression-related behavior and the functional defects in GABAergic and glutamatergic synaptic transmission of γ2 ^+/−^ mice can be reversed for a prolonged period with the rapid-acting antidepressant ketamine ^46^ (see below). Such defects in functional neural connectivity and their rescue by antidepressant therapies represent functional hallmarks of MDD ^47, 48^. Importantly, chronic treatment of γ2 ^+/−^ mice with the norepinephrine (NE) reuptake inhibitor desipramine is able to similarly normalize the behavior of γ2 ^+/−^ mice along with normalization of HPA axis function in these mice ^40^. Chronic stress–induced or optogenetic activation of NE neurons of the locus coerulus (LC) that project to dopaminergic (DA) neurons in the ventral tegmental area (VTA) mediates resilience to chronic social defeat stress of mice ^49^. In the VTA, LC-derived NE acts through α1- and β3-adrenergic receptors to induce homeostatic alterations of ion channel expression in DA neurons that contribute to stress resilience. Accordingly, we speculate that hyperexcitability of LC NE neurons in γ2 ^+/−^ mice facilitates the antidepressant action of NE reuptake inhibitors to induce slow homeostatic changes in DA neurons that underlie stress resilience. Thus, both conventional antidepressants and ketamine can act to overcome genetic (that is, hard-wired) defects in GABAergic synaptic transmission, albeit by entirely different mechanisms. Moreover, directly and deliberately increasing the excitability of certain subsets of GABAergic interneurons in mice has robust anxiolytic and antidepressant-like behavioral and biochemical consequences ^50^. Collectively, these findings lend strong support to the GABAergic deficit hypothesis of MDD and suggest that certain (but not all) agents that enhance GABAergic inhibition may have antidepressant properties ^8, 9^. For example, benzodiazepines, which act as positive allosteric modulators of GABA _A_ receptors and are first-line treatments for anxiety disorders, have only limited efficacy as antidepressants ^51^, even though they are often used to augment conventional antidepressants and to treat comorbidities of MDD, such as anxiety and insomnia ^52– 55^. Limited antidepressant efficacy of benzodiazepines may be due to tolerance, which is thought to involve chronic drug-induced degradation of major subsets of GABA _A_ receptors and corresponding loss of inhibitory synapses ^56, 57^. However, two negative allosteric modulators of α5-GABA _A_ receptors—L-655,708 and MRK-016—have been shown to exhibit rapid antidepressant-like activity in a chronic stress model of rodents ^44, 58^. Notably, in contrast to benzodiazepines and comparable to ketamine (discussed in the following), these agents act by transient disinhibition of neural circuits, which results in antidepressant-like activity in the “drug-off” situation.

The antidepressant mechanism of ketamine is unique in that it is very rapid and has the clinical benefits observed in the “drug-off” situation following a single acute dose of the drug. Recent progress in understanding of its mechanism has been thoroughly reviewed elsewhere ^59, 60^ and is only briefly recapitulated here. Key aspects of the antidepressant mechanism of ketamine are that it involves brief inhibition of GABAergic interneurons ^61^ followed by a transient surge in glutamate release ^62^ (lasting at most 1 hour) that then triggers the release of brain-derived neurotrophic factor (BDNF) ^63^ and a wave of synaptogenesis ^64^. The ensuing increase in synapse density, the corresponding restoration of neural connectivity, and normalized emotional behavior are all observed in the drug-off state and last for several days. Notably, ketamine-induced synaptogenesis and antidepressant behavioral response are drastically enhanced by GABAergic deficits, as observed in γ2 ^+/−^ mice ^46^ and also evident in animals exposed to chronic stress ^65, 66^, perhaps because neural hyperexcitability in these models facilitates the ketamine-induced glutamate surge and BDNF release. Importantly, restoration of glutamatergic synapses in γ2 ^+/−^ mice is associated with even more dramatic formation and pre- and post-synaptic potentiation of GABAergic synapses ^46^, which appears to ensure that inhibitory and excitatory synaptic transmission remain balanced. Indeed, we found no evidence in the literature that ketamine treatment triggers seizures, despite the glutamate surge and evidence of a reduced seizure threshold in patients with MDD ^67^. Similar to restoration of glutamatergic synapses, ketamine-induced strengthening of GABAergic synapses is long-lasting and observed in the drug-off situation ^46^ and temporally separated from the initial direct action of ketamine at GABAergic interneurons mentioned above. Here, we propose that *direct* and potent pharmacological enhancement of GABAergic transmission (that is, by allopregnanolone) will act accordingly to transiently dampen glutamate release and allow for lasting recovery of glutamatergic and GABAergic synaptic transmission beyond the end of treatment ( Figure 1).

## Neurosteroids differentially modulate phasic and tonic GABAergic inhibition

Neurosteroids are metabolites of cholesterol-derived steroid hormones synthesized in the brain by neurons and astrocytes. They act as potent, endogenous, positive allosteric modulators of GABA _A_ receptors and include derivatives of progesterone and deoxycorticosterone, in particular 3a,5a-tetrahydroprogesterone (3α,5α-THP; allopregnanolone), 3α,5β-tetrahydro-progesterone (3α,5β-THP; pregnanolone), and 3α,5α tetrahydrodeoxycorticosterone (3α,5α-THDOC; allotetrahydro-deoxycorticosterone).

Neurosteroids such as allopregnanolone have little effect on the rise time or the amplitude of GABA-induced synaptic currents but primarily prolong the decay kinetics of the GABA-gated ion channel ^68^, which results in an increase of the mean channel open time of the GABA-activated chloride channel and a prolonged inhibitory post-synaptic current. However, when acting on extra-synaptic receptors that are kept tonically active by ambient concentrations of GABA, the allopregnanolone-induced prolonged decay kinetic results in an increased amplitude of the tonic current. In addition to potentiation of GABA _A_ receptor channel function ^69, 70^, allopregnanolone and its synthetic derivatives may potentiate GABA transmission by promoting the cell surface expression of GABA _A_ receptors ^71^. The impact on phasic or tonic inhibition can be strikingly different because of the type of GABA _A_ receptor subtype involved. The prototypic synaptic receptor contains α1, β2/3, and γ2 subunits and is sensitive to physiological concentrations of neurosteroids ^68^. However, in certain cells, such as dentate gyrus (DG) granule cells or cerebellar granule cells, allopregnanolone at low concentrations (10–100 nM) selectively enhances tonic inhibition with little or no effect on phasic conductance. This appears to be due largely to the preponderance of highly neurosteroid-sensitive extra-synaptic δ subunit–containing receptors with the subunit combinations α4,β3,δ, and α6,β2,3,δ ^72– 74^. In addition, phosphorylation of the β3 subunit by protein kinase C appears to promote neurosteroid sensitivity of extra-synaptic receptors while limiting that of synaptic receptors ^75^. In some neurons, the strict division of GABA _A_ receptors into synaptic and extra-synaptic receptors mediating phasic and tonic inhibition, respectively, has become an oversimplification ^76^. The therapeutic action of brexanolone (peak steady-state plasma concentration of about 150 nM; see below) is likely to comprise an enhancement of both phasic and tonic inhibition.

## Downregulation of neurosteroids in affective disorders

The downregulation of neurosteroid biosynthesis has been implicated as a possible contributor to various psychiatric conditions, as shown in a number of clinical trials. In patients with MDD, allopregnanolone and pregnanolone were decreased in cerebrospinal fluid (CSF) ^77^ and in plasma ^78, 79^. Plasma allopregnanolone was similarly decreased in postpartum “blues” ^80^ (but see below), post-traumatic stress disorder ^81, 82^, schizophrenia negative symptomatology ^83^, pain ^84^, and pharmacologically induced panic attacks ^85^ but did not reach significance in general anxiety disorder ^86^. Conversely, the 3β isomer of allopregnanolone antagonizes GABA _A_ receptor function ^87^ and is increased in panic attacks ^88^. Based on studies of postmortem brain, changes in the neurosteroid synthesis pathways were also proposed to contribute to the pathologies of neurodegenerative and inflammatory diseases (Alzheimer’s and Parkinson’s disease and multiple sclerosis) ^89^.

## Stress-induced behavior linked to downregulation of neurosteroids

Because chronic stress is a major risk factor for depression, the influence of chronic stress on neurosteroids has become a major focus. In striking contrast to acute stress, which increases allopregnanolone levels ^89, 90^, chronic stress and pharmacological induction of panic attacks ^84^ result in reduced levels of neurosteroids. In animal models of chronic stress, the concentration of allopregnanolone was decreased in serum ^91– 93^ and in selected corticolimbic brain areas ^94^. This decrease was attributed to stress-induced downregulation of the 5α-hydroxysteroid-dehydrogenase, the rate-limiting enzyme in the synthesis of allopregnanolone ^92, 95, 96^. Moreover, the stress-induced reduction of allopregnanolone was associated with heightened depressive/anxiety-like behavioral phenotypes, increased fear and aggression behavior, dysregulation of the HPA axis ^92, 94, 97^, and impaired adult hippocampal neurogenesis ^98, 99^.

## Allopregnanolone ameliorates anxiety- and depression-related behavior

Administration of allopregnanolone either before or after a period of chronic stress was able to alleviate the symptoms of depressive/anxiety behavior, prevent or normalize HPA axis dysfunction, and restore neurogenesis and cognitive deficits in transgenic mouse models of Alzheimer’s disease ^99– 101^. Furthermore, allopregnanolone and pregnanolone produced anxiolytic-like effects in various animal models of anxiety ^102^. Micro-infusion of allopregnanolone identified the amygdala as being relevant for anxiolysis ^103^ and both the hippocampus and amygdala for overcoming learned helplessness ^104^. These results support a role of allopregnanolone in ameliorating symptoms of depression and anxiety and thereby support the view that a pathological deficit of GABAergic transmission contributes to these disorders ^8, 9, 105^.

## Classic antidepressants normalize neurosteroid levels in depression

With allopregnanolone being able to overcome depressive-like behavior, the question arose whether classic antidepressant drugs would act via an enhancement of neurosteroid levels. In animal models of depression, multiple antidepressants (fluoxetine, fluvoxamine, sertraline, desipramine, venlafaxine, and paroxetine) normalized corticolimbic levels of allopregnanolone concomitant with reduced anxiety-like, fear, and aggression behavior ^83, 92, 106, 107^. This effect, as shown for selective serotonin reuptake inhibitors (SSRIs), was independent of their ability to inhibit serotonin reuptake ^108– 110^ and in the case of fluoxetine is thought to involve inhibition of a microsomal dehydrogenase that oxidizes allopregnanolone to 5α-dihydroprogesterone ^111^.

These findings gave reason to test whether the clinical effectiveness of SSRIs was linked to normalizing the allopregnanolone level. Indeed, in patients with unipolar depression, the decreased allopregnanolone level, measured in CSF, was normalized after 8 to 10 weeks of treatment with fluoxetine or fluvoxamine and correlated with an improved symptomatology ^77^. This finding was extended to a range of antidepressants (SSRIs and tricyclic antidepressants), which normalized plasma allopregnanolone levels concomitant with an improvement of depression ^109^. A normalization of plasma allopregnanolone and pregnenolone was also seen following 3 weeks of mirtazapine treatment in patients with major depression ^79^. Notably, the mirtazapine-induced maximal increases in these neurosteroids preceded their maximal clinical effects by about 2 weeks, suggesting that they are part of the pharmacological response mechanism rather than a subsequent measure of clinical improvement. These pre-clinical and clinical findings support the view that the neurosteroidogenic action of SSRIs may constitute a major part of their therapeutic effectiveness in patients with depressive disorders.

## Postpartum risk of depression

Postpartum depression (PPD) is an important public health issue as it affects women at a highly vulnerable time and can affect the cognitive and emotional development of the child ^112^. The risk of depression in women becomes significantly increased during the postpartum period, and nearly 20% of mothers have PPD, which is frequently preceded by antenatal anxiety- and depression-related symptoms or chronic stress as the strongest predictors ^113– 115^. PPD is frequently attributed to a maladaptation to peripartum fluctuations in reproductive hormone levels during pregnancy and the postpartum period ^116, 117^. Plasma allopregnanolone concentrations rise in parallel with progesterone throughout pregnancy, reaching the highest level in the third trimester and decreasing abruptly after childbirth ^80^.

Nevertheless, peripartum changes in gonadal hormones affect the emotional brain in vulnerable women. PPD was characterized by abnormal activation of the same brain regions implicated in non-puerperal major depression ^118^. The resting-state functional connectivity within corticolimbic regions implicated in depression was attenuated compared with healthy postpartum women ^114^. Similarly, emotionally normal (euthymic) women with a history of PPD showed stronger signs of depression than controls in tests of withdrawal from supra-physiological gonadal steroid levels ^119^. Additional factors that have been implicated in the pathophysiology of PPD include the lactogenic hormones oxytocin and prolactin, thyroid function, and a hyperactivity of the HPA axis ^117^. As outlined below, the potential importance of GABA _A_ receptor plasticity in PPD has been derived largely from animal studies ^120^.

## Animal models of postpartum depression and GABAergic impact

Rodent models suggest that both phasic and tonic GABAergic inhibition in the brain are decreased during pregnancy in parallel with a decrease in GABA _A_ receptor expression as shown for the GABA _A_ receptor γ2 and δ subunit in mouse and rat hippocampus ^121^. Within days after parturition, GABAergic transmission and the level of GABA _A_ receptor expression rebound to control levels ^121, 122^. This fluctuation in receptor expression is considered to be a homeostatic response to the elevated levels of pregnenolone and allopregnanolone in plasma and brain during rodent pregnancy and their rapid return to control levels postpartum ^122, 123^.

A transgenic animal model of PPD supports the view that the pathophysiology of PPD may be related to a deficit of GABA _A_ receptor plasticity. Mice that lacked the GABA _A_ receptor δ subunit partly or fully ( *Gabrd* ^+/−^ and *Gabrd* ^−/−^) exhibited PPD-like behavior (reduced latency to immobility in the Porsolt forced swim test and reduced sucrose preference) and abnormal maternal behavior (reduced nesting behavior and pup care) ^121^. Remarkably, the mice were behaviorally unremarkable until an animal was exposed to pregnancy and the postpartum state. Thus, reproductive events unmask the genetic susceptibility to affective dysregulation. The abnormal postpartum behavior in *Gabrd* ^+/−^ mice was ameliorated by THIP, a GABA analogue with preferential affinity to GABA _A_ receptors containing the δ subunit ^121^ and the neuroactive steroid SGE-516 ^115^.

Another animal model suggests that the dysregulation of the HPA axis is sufficient to induce abnormal postpartum behavior ^115^. CRH neurons in the paraventricular nucleus (PVN) of the hypothalamus govern the HPA axis and are critical for mounting the physiological response to stress. Chemogenetic activation of CRH neurons in the PVN was sufficient to induce abnormal postpartum behavior. Similarly, when GABAergic currents were reduced selectively in CRH neurons (KCC2/Crh mice), a depression-related phenotype and a deficit in maternal behavior were apparent in the postpartum period. In wild-type mice, the stress-induced activation of the HPA axis and the corresponding elevation of circulating corticosterone are normally blunted during pregnancy and postpartum ^124^. The inability to blunt this stress-induced HPA axis activation in this model is thought to contribute to PPD. The neuroactive steroid SGE-516 ameliorated the behavioral deficits caused by the dysregulation of the HPA axis ^115^.

## Brexanolone in the treatment of postpartum depression

In line with the evidence described above, brexanolone, an intravenous formulation of allopregnanolone, underwent clinical tests to treat PPD. In a double-blind, randomized, placebo-controlled phase II trial, female in-patients with PPD (n = 21) received a 60-hour continuous infusion of brexanolone at a dose of up to 60 μg/kg per hour or placebo. Women who received treatment, in comparison with those who received placebo, had a significant and clinically meaningful reduction in mean total score on the 17-item HAMILTON Rating Scale for Depression (HAM-D) at the 60-hour time point ^125^. In a subsequent phase III study with 246 patients, performed in two parts using the same design but two doses of drug, the HAM-D score was again significantly and clinically meaningfully decreased. The HAM-D total score mean reductions versus placebo were −5.5 and −3.7 points for the high and low dose, respectively (90 and 60 μg/kg per hour), and dizziness and somnolence were potential side effects ^126^. Notably, brexanolone is very rapidly cleared from plasma, which explains the need for continuous drug infusion ^127^. The higher dose explored above (90 μg/kg per hour) results in peak steady-state plasma concentrations of allopregnanolone (50 ng/mL) equivalent to those naturally reached in the third trimester of pregnancy ^128^. The antidepressant drug effect was shown to become significant within 48 hours of drug infusion for both doses. Moreover, the mean reduction in HAM-D total scores observed for the high-dose treatment at the end of the study (day 30) was similar in magnitude to that observed at the end of the 60-hour infusion. Thus, in the context of PPD, brexanolone showed a rapid mode of action that is reminiscent of that of ketamine in MDD and appears to result in durable clinical improvement ( Figure 1). Brexanolone was very recently approved for the treatment of PPD ^129^.

Although a decrease in serum allopregnanolone (but not progesterone) was reported in one study in women with postpartum “blues” ^80^, there is no consistent evidence of abnormal basal circulating levels of allopregnanolone in PPD ^114, 116, 117, 130^. With allopregnanolone levels being normal, the antidepressant action of brexanolone is attributed to an enhancement of GABA _A_ receptor function, thereby supporting the GABAergic deficit hypothesis of depression. In addition, emerging evidence suggests that allopregnanolone has anti-inflammatory effects *in vitro*, a property that could contribute to its antidepressant activity *in vivo* ^78^. The antidepressant properties of brexanolone in PPD may be applicable to other forms of depression that are less clearly linked to altered neurosteroid physiology and that may be associated with defects in phasic rather than tonic GABAergic inhibition ^113, 114, 118^. Indeed, emerging evidence suggests that the anxiolytic effects of endogenous neurosteroids (even at their natural physiological concentrations) are mediated in part by α2 subunit–containing synaptic GABA _A_ receptors ^42^. Moreover, a synthetic derivative of allopregnanolone (zuranolone, SAGE-217) is currently in phase 3 clinical development for PPD and MDD ^131^. Notably, potentiation of GABA transmission by brexanolone and potentially also zuranolone, unlike SSRIs that exhibit therapeutic delays of weeks or months, confers rapid and lasting antidepressant effects that are observed in the drug-off situation, reminiscent of mechanisms of ketamine. Interestingly, since allopregnanolone promotes proliferation of progenitor cells and restores neurogenesis in disease states ^99– 101, 132^, it may also support neurogenesis-dependent resilience behavior, as outlined below.

## Resilience due to changes in neural circuits

Studies of stress resilience have opened up a fundamentally new way of understanding an individual’s response to adverse life events such as trauma, tragedy, and chronic stress and its ability to avoid deleterious behavioral changes such as anxiety disorders, post-traumatic stress disorder, or depression. Although resilience, as defined in humans, is difficult to relate to animal studies, animal models are indispensable in the search for biological determinants of resilience (that is, protective changes that occur in resilient animals). This is all the more important as mechanisms that promote resilience to stress hold the promise of enabling the development of more efficacious antidepressant therapies.

After chronic social defeat stress, about 40% of the stressed mice do not exhibit social avoidance or anhedonia in subsequent testing ^133, 134^. This is interpreted as resilience behavior and is associated with many distinct changes, particularly in the brain’s reward regions ^133– 135^. These changes include homeostatic adaptations of dopamine neurons in the VTA that prevent chronic stress–induced aberrant hyperexcitability of these cells ^49, 136, 137^, the induction of immediate early gene products in the medial prefrontal cortex (mPFC) ^138^, the sustainment of GABAergic inhibition and reduction of excitatory activity in the nucleus accumbens ^139^, the prevention of spine density loss in the mPFC and hippocampus ^140^, and epigenetic genomic changes that promote resilience in later life because of greater maternal care ^141, 142^.

## Adult neurogenesis linked to resilience via GABAergic inhibition

More recently, adult neurogenesis in the DG of the hippocampus has been implicated in conferring resilience to the detrimental depressive-like consequences of chronic stress exposure of mice ^143^. Chemogenetic inhibition of immature adult-born neurons in ventral DG (vDG) *in vivo* promoted susceptibility to social defeat stress. In contrast, increasing neurogenesis by inducible deletion of the proapoptotic gene *Bax* (iBax mice) selectively from adult neural progenitor cells conferred resilience to chronic stress as measured by the time spent socially interacting with a novel mouse and the time spent exploring the center in an open field. On the cellular level, a subset of mature DG cells was identified as stress-responsive cells that were active preferentially during attack (17% of cells on defeat day 1 to 34% of cells on defeat day 10). The activity of the stress-responsive mature cells was decreased when neurogenesis was increased. Thus, immature adult-born granule cells inhibit mature stress-responsive granule cells in the vDG, which protects the animals from chronic stress–induced depressive and anxiety-like consequences. The inhibition of mature granule cells by immature adult-born cells is likely to involve activation of hilar GABAergic interneurons that are known to confer a strong inhibitory influence on mature granule cells ^144^. Neurosteroids are predestined to inhibit DG granule cells because of their high level of expression of δ subunit–containing GABA _A_ receptors, which are highly neurosteroid-sensitive ^68, 72– 74^.

The finding that GABAergic interneuron activity can support resilience may give rise to a potential GABAergic hypothesis of resilience, which conceivably is independent of the prerequisite of adult neurogenesis. Decreased GABAergic interneuron activity would be expected to reduce resilience behavior whereas enhancing GABAergic transmission would sustain it. The neurosteroid allopregnanolone appears to be a case in point. Its clinical effectiveness as an antidepressant in treating PPD might be supported, at least in part, by its ability to foster resilience.

## Outlook

The demonstration of the clinical effectiveness of allopregnanolone in PPD lends new support to the GABAergic deficit hypothesis of MDD. This finding bodes well for further investigations of ligands for the neurosteroid site as a new class of drugs for affective disorders. To achieve this goal, a differentiation of GABA _A_ receptors beyond that achieved by allopregnanolone is required. The recent molecular x-ray resolution of the neurosteroid binding domain is an essential step forward. In chimeric homopentameric GABA _A_ receptor constructs, the neurosteroid THDOC was bound at the bottom of the transmembrane domain across each of the subunit interfaces ^145– 147^ and similar findings are expected for heteropentameric GABA _A_ receptors ^145, 148^. These studies provide a structural framework for the development of more selective ligands acting at the neurosteroid site for the treatment of affective disorders, including PPD and MDD, but also pain and epilepsy.
